# Limited receipt of support services among people with mild‐to‐moderate dementia: Findings from the IDEAL cohort

**DOI:** 10.1002/gps.5688

**Published:** 2022-02-06

**Authors:** Jayden O. van Horik, Rachel Collins, Anthony Martyr, Catherine Henderson, Roy W. Jones, Martin Knapp, Catherine Quinn, Jeanette M. Thom, Christina Victor, Linda Clare, IDEAL Programme Team

**Affiliations:** ^1^ REACH: The Centre for Research in Ageing and Cognitive Health University of Exeter Exeter UK; ^2^ Clinical Trials Unit University of Exeter Medical School Exeter UK; ^3^ Care Policy and Evaluation Centre London School of Economics and Political Science London UK; ^4^ RICE: The Research Institute for the Care of Older People Royal United Hospitals Bath UK; ^5^ The Centre for Applied Dementia Studies University of Bradford Bradford UK; ^6^ Wolfson Centre for Applied Health Research Bradford UK; ^7^ School of Medical Sciences University of New South Wales Sydney Australia; ^8^ College of Health, Medicine and Life Sciences, Department of Health Sciences, Brunel University London Uxbridge UK; ^9^ NIHR Applied Research Collaboration South‐West Peninsula Devon UK

**Keywords:** Alzheimer's disease, healthcare guidelines, policy, quality of life

## Abstract

**Background:**

Global initiatives that promote public health responses to dementia have resulted in numerous countries developing new national policies. Current policy guidelines in England, for example, recommend that people diagnosed with mild‐to‐moderate dementia receive information and psychosocial interventions to improve their ability to ‘live well’. However, it remains unclear to what extent these recommendations are being achieved.

**Methods:**

Self‐reported information from 1537 people living with dementia and informant‐reported information from 1277 carers of people living with dementia was used to quantify receipt of community‐based dementia support services, including health and social care services provided by statutory or voluntary‐sector organisations, in Britain from 2014 to 2016. Demographic factors associated with differences in receipt of support services were also investigated to identify particularly vulnerable groups of people living with dementia.

**Results:**

Both self‐ and informant reports suggested that approximately 50% of people living with dementia received support services for dementia. Receipt of support services was lower among people living with dementia who are older, female, and have fewer educational qualifications. Receipt of support services also differed according to diagnosis and carer status, but was unrelated to marital status.

**Conclusions:**

Limited receipt of dementia support services among people living with dementia in Britain provides a baseline to assess the efficacy of current policy guidelines regarding provision of information and support. Targeted efforts to facilitate receipt of support services among the particularly vulnerable groups identified in the current study could improve the efficacy of dementia support services both in Britain and internationally, and should inform policy development.

## Key points

- In Britain between 2014 and 2016, approximately 38% (range = 29–54%) of people living with dementia and 57% (range = 33–75%) of informants from the IDEAL cohort reported receipt of community‐based support services to help with their dementia diagnosis.
- Receipt of dementia support was lower among people living with dementia who were older, female, had fewer educational qualifications, and who were diagnosed with Alzheimer's disease rather than Parkinson's disease dementia or dementia with Lewy bodies (DLB).
- Targeted efforts that facilitate receipt of dementia support services among particularly vulnerable groups could improve the efficacy of national policy and reduce inequality.

## 1 INTRODUCTION

Dementia affects approximately 50 million people worldwide and this number is predicted to increase to 152 million by 2050.^1^ National responses are therefore required to improve the lives of people living with dementia and their carers, and reduce the potential impact of dementia on communities and countries. Consequently, the World Health Organization (WHO) has developed a global action plan that urges governments to develop national policies on dementia, including actions to meet the care and support requirements of people living with dementia and their carers by 2025.^2^ Numerous countries are working towards meeting the targets of the WHO global plan.^3^, ^4^ In England, for example, current National Institute for Health and Care Excellence (NICE) guidelines recommend that, in addition to having the opportunity to receive evidence‐based interventions as required to address specific clinical symptoms and needs, people living with dementia are provided with support services that include: (i) accessible information that is relevant to their circumstances and the stage of their condition; (ii) a single named health or social care professional who is responsible for coordinating their care; and (iii) support to engage in activities that reflect personal preferences and needs.^5^ Yet, the extent to which national policies have improved the receipt of support services among people living with dementia, and whether there are inequalities in the receipt of support services, currently remains unclear.

People living with dementia who obtain access to dementia‐specific services early in their dementia journey can improve their ability to live independently^6^, ^7^ and delay the potential need for institutional care.^8^ However, receipt of community‐based dementia support services, including health and social care services provided by statutory or voluntary‐sector organisations, is often limited^9^, ^10^, ^11^ and potentially fails to meet the needs of people living with dementia and their carers.^12^ Although access to community‐based support services is influenced by the availability of publicly‐funded health resources, access to public or private transportation, and whether or not these services are considered helpful,^13^ there are a number of additional barriers that further impede the receipt of formal community‐based care, including inaccessibility of information.^13^, ^14^, ^15^ Moreover, specific groups of people living with dementia and their carers, such as those with fewer educational qualifications,^16^, ^17^, ^18^ married couples,^19^ and those with insufficient transport^20^ may also be disproportionately less likely to use support services.

To address the extent to which national healthcare guidelines on dementia are currently being met, it remains necessary to establish a baseline from which improvements can be subsequently assessed. Baseline data from the Improving the experience of Dementia and Enhancing Active Life (IDEAL) cohort of people with mild‐to‐moderate dementia and their carers living in Britain^21^, ^22^ was therefore used to address the following two questions: (1) What is the extent to which people living with dementia and carers report they receive dementia support services? and (2) are there variations in reported receipt of support services among groups of people living with dementia that share particular demographic characteristics? Findings from this study can provide a foundation to assess improvements in receipt of support for people with dementia in Britain and compare progress cross‐nationally, which can then inform policy development.

## 2 METHODS

### 2.1 Participants

Self‐reported information from 1537 people living with dementia and, in the case of 1277 of them, informant‐reported information from carers, was used in this study. Informant reports, where available, were compared with those obtained from people living with dementia and the extent of agreement was quantified.^23^ Data were obtained from the baseline (Time 1; T1) assessment of the IDEAL cohort, collected between 2014 and 2016.^21^ All participants with dementia were recruited through the UK National Health Service (NHS), had a clinical diagnosis of mild‐to‐moderate dementia, and were living in the community in Britain (i.e. in England, Scotland or Wales). Trained researchers collected data over the course of three separate visits to participants' homes. Caregivers self‐completed the informant questionnaires, while the researcher interviewed the person with dementia. Full inclusion, exclusion and consent criteria are provided in the IDEAL study protocol.^21^ The IDEAL study was approved by the Wales Research Ethics Committee 5 (reference 13/WA/0405) and the Ethics Committee of the School of Psychology, Bangor University (reference 2014–11684), and is registered with UKCRN, registration number 16593.

### 2.2 Post‐diagnostic and ongoing support services

People living with dementia and informants in the IDEAL cohort were asked a set of “support services questions”. These questions were developed with input from two different groups: the IDEAL programme Project Advisory Group; and the IDEAL programme involvement group, comprised of people with dementia and carers, known as the ALWAYs (Action on Living Well: Asking You) Group.^24^

The questions sought to identify whether people living with dementia and their carers in Britain (1a) had a named health professional that they could contact at any time, and if so (1b) whether that health professional was in place due to the dementia diagnosis, (2) had received information or educational materials about the condition, (3) had attended psychosocial interventions, and (4) had independently sought information about their dementia diagnosis. A verbatim description of questions presented to participants, including examples, is presented in Table S1. All questions elicited “Yes” or “No” responses.

### 2.3 Demographic variables

The following demographic variables were included for people living with dementia: (1) Sex: male, female; (2) Age (years); (3) Marital Status: married/partnership/cohabiting; divorced/legally separated; widowed; single; (4) Carer Status: spouse/partner; family/friend; no carer involved; (5) Educational Group: no qualification; school leaving certificate at age 16; school leaving certificate at age 18; university; (6) Diagnosis: Alzheimer's disease (AD); vascular dementia; Mixed AD/vascular dementia; frontotemporal dementia; Parkinson's disease dementia (PDD); dementia with Lewy bodies (DLB); unspecified/other.

### 2.4 Statistical analysis

All analyses were done on Version 5, the most recent version to‐date, of the IDEAL dataset. Generalised linear models (GLM) from the lme4 package^25^ in R V1.0.143^26^ were used for all analyses. To determine whether differences in demographic variables (predictor variables) were associated with information about receipt of support services elicited from people living with dementia and informants (binary response variables), GLM with a binomial error distribution and logit link function was employed and included all demographic variables in a full backwards stepwise model deletion using the drop1 (Model1, test = “Chi”) function to select the best fitting minimal model with the lowest AIC values. The first level reported within each variable reflects the reference to which subsequent differences between groups were compared (Table 1). Informant sex and age are presented (Table 1) but were not included in the analyses. Not all people living with dementia or informants completed all questions. Therefore, individuals with missing data were excluded from each respective analysis for accurate model comparisons. Tables of demographic variables that were retained in each minimal model are reported in Supplementary Material (SM).

**TABLE 1:** Sample size and percent composition of people living with dementia (PwD) for each demographic measure obtained from the IDEAL cohort, including sex, age in years ± SEM (Standard Error), and carer status of informants. Participants Mini‐Mental State Examination (MMSE) score ± SEM is also presented

Demographic measures	Levels	n	%
PwD			
MMSE	n/a	23.22 ± 0.1	n/a
Sex	Male	865	56.28
	Female	672	43.72
Age	Male	76.0 ± 0.3	
	Female	76.9 ± 0.3	
Marital status	Married/Partnership/Cohabiting	1152	75
	Divorced/Legally separated	92	6
	Widowed	266	17
	Single	27	2
Educational group	No qualifications	427	28
	School leaving certificate at age 16	271	18
	School leaving certificate at age 18	521	34
	University	309	20
	Missing	9	1
Diagnosis	Alzheimer's disease	851	55.37
	Vascular dementia	170	11.00
	Mixed Alzheimer's disease/vascular dementia	324	21.08
	Frontotemporal dementia	54	3.50
	Parkinson's disease dementia	44	2.90
	Dementia with Lewy bodies	53	3.40
	Unspecified/other	41	2.70
Informant			
Sex	Male	394	30.83
	Female	883	69.17
Age	Male	73.0 ± 0.5	
	Female	67.5 ± 0.4	
Carer status	Spouse/Partner	1042	68
	Family/Friend	235	15
	No carer involved in study	260	17

## 3 RESULTS

Demographic characteristics of people living with dementia are presented in Table 1. A summary of responses to support services questions for people living with dementia, informants, and dyads, including number of Yes responses to each respective question, agreement of dyadic responses, and percentages of responses relative to overall sample size, is presented in Table 2. A summary of significant demographic measures generated from all people living with dementia and informant minimal models (GLM) that predict receipt of support services is presented in Table 3.

**TABLE 2:** Total number of responses (sample size) and proportion of Yes responses to support services questions from people living with dementia (PwD), informants, and dyads. Percentage values of totals are presented in parentheses. Agreement of dyadic responses are also presented

Questions:	1a. Named health professional	1b. Health professional in place due to dementia diagnosis	2. Received information or educational materials	3. Interventions	4. Independently sought information
Total responses (%)					
PwD	1511 (98)	424 (97)^a^	1379 (90)	1374 (89)	1376 (90)
Informant	1205 (94)	465 (99)^a^	1242 (97)	1231 (96)	1236 (97)
Dyad	1189 (93)	209 (98)^a^	1121 (88)	1111 (87)	1114 (87)
Yes responses (%)					
PwD	438 (29)	227 (54)	652 (47)	404 (29)	435 (32)
Informant	468 (39)	349 (75)	991 (74)	408 (33)	760 (62)
Agreement (%)					
Dyad	792 (67)	134 (64)	612 (55)	812 (73)	596 (54)

^a^ Denotes percentages relative to Yes responses in the preceding question.

**TABLE 3:** Summary of significant (Sig; *p* < 0.05) demographic measures from people living with dementia and informant information about support services questions retained in minimal models (Generalised linear models (GLM)). The direction of each significant measure is reported in the corresponding results section for each respective analysis, and analyses for both significant and non‐significant measures are presented in the results tables within the Supplementary Materials. Marital Status was non‐significant for all models and is therefore omitted

Questions	Sex	Age	Carer status	Educational group	Diagnosis
People living with dementia					
1a. Named health professional	Sig.	Sig.		Sig.	Sig.
1b. Health professional in place due to dementia diagnosis		Sig.			
2. Received information or educational materials		Sig.			Sig.
3. Interventions	Sig.	Sig.	Sig.		
4. Independently sought information	Sig.	Sig.		Sig.	Sig.
Informant					
1a. Named health professional		Sig.	Sig.		Sig.
1b. Health professional in place due to dementia diagnosis			Sig.		Sig.
2. Received information or educational materials	Sig.				Sig.
3. Interventions				Sig.	Sig.
4. Independently sought information	Sig.	Sig.	Sig.	Sig.	

### 3.1 What is the extent to which people living with dementia and carers report they receive dementia support services?

Across all support services questions, 38% (range = 29–54%) of people living with dementia reported receipt of support services, compared to 57% (range = 33–75%) of carers who reported that people with dementia received these services (Table 2). Although people living with dementia reported lower receipt of support services than informants, dyadic agreement was above 50% for all support services questions (overall mean = 63%, range = 54–73%; Table 2).

### 3.2 Are there variations in reported receipt of support services among groups of people living with dementia that share particular demographic characteristics?

#### 3.2.1 Named health professional

People living with dementia were more likely to report that a named health professional was available to contact should they need support at any time if they were male, younger, had a school leaving certificate at age 16, or were diagnosed with PDD or DLB, than people living with dementia who were female, older, had no educational qualifications, or were diagnosed with AD (Table S2). Informants reported that they were more likely to consider that a named health professional was available to contact should they need support at any time if the person living with dementia was younger, diagnosed with PDD rather than AD, or had a carer who was a family member or friend rather than a spouse or partner (Table S2). There were no other significant demographic predictors (Table S2).

#### 3.2.2 Health professional in place due to dementia diagnosis

Of those people living with dementia and informants who reported that a named health professional was available to contact if they needed support at any time (Question 1a; Table S1), younger people living with dementia were more likely to consider that the availability of the health professional was due to the diagnosis than older people living with dementia (Table S3). Informants were more likely to report that a health professional was available because of the dementia diagnosis if the carer was a spouse or partner, or if the person living with dementia was diagnosed with AD, than if the carer was a family member or friend, or if the person living with dementia was diagnosed with PDD (Table S3). No other significant demographic predictors were identified (Table S3).

### 3.3 Received information or educational materials

People living with dementia were more likely to report that they received information relating to the diagnosis if they were younger, or diagnosed with PDD, than people living with dementia who were older or diagnosed with AD (Table S4). Informants of men living with dementia, and those diagnosed with AD, were more likely to report that they had received information to help with their diagnosis, than women living with dementia or people who were diagnosed with PDD (Table S4). No other significant demographic predictors were identified (Table S4).

### 3.4 Psychosocial interventions

People living with dementia were more likely to have received an intervention if they were male, younger, or their carer was their spouse or partner, than people living with dementia who were female, older, or did not have a carer (Table S5). Informants reported that people living with dementia were more likely to have received psychosocial interventions if they had left school at 18 with a certificate than if they had no educational qualifications (Table S5). Informant reports also indicated that people living with dementia were more likely to have received psychosocial interventions if they were diagnosed with Mixed AD/vascular dementia rather than AD, or AD rather than PDD. No other significant demographic predictors were identified (Table S5).

### 3.5 Independently sought information

People living with dementia were more likely to independently seek out information to help with the diagnosis if they were male, younger, had an educational qualification, or were diagnosed with PDD or DLB, than people living with dementia who were female, older, had no educational qualifications, or were diagnosed with AD (Table S6). Informants of men living with dementia were more likely to report that they had independently sought information to help with their diagnosis, than women living with dementia (Table S6). Informants were also more likely to independently seek information to help the person living with dementia if they themselves were a family member or friend than a spouse or partner, or if the person living with dementia was younger, or had a university education, than if the person living with dementia was older, or had no qualifications or school leaving certificate at age 16 or 18 (Table S6). There were no other significant demographic predictors (Table S6).

## 4 DISCUSSION

To the best of our knowledge, this is the first study to investigate the extent to which people living with dementia and their informants in Britain report receiving support services, and to consider the underlying inequalities that may influence differences in receipt of these services. Findings obtained from self‐ and informant reports of people living with mild‐to‐moderate dementia and their carers in 2014–2016 show low rates of receipt of dementia support services, with inequalities among groups defined by different demographic characteristics. People with dementia who were female, older, and had fewer educational qualifications received fewer dementia support services.

Findings from the current study confirm responses from a survey of 1013 GPs indicating that people living with dementia in England received little post‐diagnostic support.^27^ Low receipt of support services among people living with dementia may indicate that services are unavailable, or that they are available but not used, possibly because they are not known about, are considered unhelpful, or are impractical to access. Receipt of support services may therefore be influenced by either intrinsic factors that are mediated at a personal level, such as age or sex, or extrinsic factors that reflect deficits in statutory or policy guidelines, such as support that meets the requirements of people with a specific type of dementia, such as one of the rarer sub‐types. Although the IDEAL datasets preclude differentiating between unavailability and underutilisation of support services, these data provide helpful insights into the way in which receipt of community‐based support services may be influenced by both intrinsic and extrinsic factors.

Particular groups of people living with dementia received fewer support services than others. Findings suggest that as age increases receipt of support services decreases; however, it should be noted that the effect size of this relationship indicates that these differences are small. There was lower receipt of support services among women living with dementia than among men living with dementia. Although parallel findings are reported among carers of people living with dementia who are older women,^28^ female carers are also said to attend more support services, such as Dementia Cafés, than male carers.^18^ Together, these findings highlight the importance of considering how sex differences influence receipt of support services among people living with dementia and their carers, and reiterate the growing concern that policies to promote gender awareness and reduce inequality in dementia care are not being met.^29^

Low receipt of support services was also identified among spousal or partner dyads. These findings parallel reports in the carer literature, which have been proposed to result from feelings of personal responsibility in providing care,^19^, ^30^ but may also suggest that it is more difficult for spousal carers to receive or access available support, or alternatively that these dyadic relationships are more robust to caregiving requirements. People living with dementia attended more psychosocial interventions if cared for by a spouse or partner than if they had no carer involved, reiterating the important role carers may play in facilitating the use of support services. Findings also suggest that fewer years of formal education correspond with lower receipt of support services among people living with dementia and their carers.^16^, ^17^, ^18^ However, some studies report no influence of educational level and utilisation of community support services among carers of people living with dementia,^9^, ^31^ suggesting that the relationship between educational attainment and receipt of support services requires further attention.

Above, we highlight intrinsic factors associated with low receipt of support services. However, extrinsic factors may also play a role. Extrinsic differences may reflect disparity in the availability of public health resources. For instance, people diagnosed with AD generally received fewer support services than people diagnosed with PDD or DLB. This may be because people with PDD and DLB have poorer QoL and ‘living well’ scores – as do their carers – compared to people with AD, VaD and FTD.^32^ Movement disabilities associated with PDD and DLB may also be more likely to require specialised symptom management than difficulties associated with AD, therefore resulting in more frequent provision of support services.^33^ Moreover, people with PDD may have received support from specialist Parkinson's disease services prior to developing dementia, which may improve subsequent receipt of support compared to people with AD, for whom support may be more limited.

Above, several limitations to this study were highlighted that constrain interpretation about why receipt of services is disproportionately low among particular groups of people living with dementia. However, the current study includes perspectives from a large cohort of people living with dementia in Britain that was broadly representative of the population attending NHS memory assessment services; for example, the distribution of dementia diagnoses was consistent with available estimates of population values.^27^ Consequently, interpretations of responses are constrained by practical limitations that result from a trade‐off between the depth of information included in the support services questions and the breadth and diversity of participants included in this study. Reasonable levels of agreement in dyadic responses of receipt of support services among people living with dementia were obtained (Table 3). However, aside from age, demographic measures associated with the subjective receipt of support services were rarely consistent between self‐ and informant ratings. For example, self‐ratings suggested that people diagnosed with PDD received information or educational materials to help with their diagnosis, whereas informant ratings suggested that they had not received such information. Although these inconsistences suggest that the findings be interpreted with caution, they also highlight important differences between the subjective perceptions of people living with dementia and their informants.^34^

To improve receipt of dementia support services among people living with dementia, it is considered important to facilitate early contact with a named healthcare professional who can provide tailored advice and support at any time.^5^, ^8^, ^13^ Moreover, it remains essential to actively promote the availability of support services and provide effective channels to which these services can be received. This may include making resources available in multiple formats rather than exclusively online and targeting particularly vulnerable groups, such as older people living with dementia, females and those with fewer educational qualifications. Subsequent studies could also evaluate whether low receipt of support is due to service unavailability, or because existing services are considered unhelpful or too impractical to access.

In conclusion, findings suggest that at the time of data acquisition, under a free universal public healthcare system in Britain, national policy guidelines in place at that time^35^, ^36^, ^37^ to address global initiatives on public health targets associated with dementia^38^, ^39^ were only being partially met. This study identified disproportionately low receipt of support services among people living with dementia who are women, older, and have fewer educational qualifications and highlights the need for targeted efforts to reduce this inequality. These findings provide an important baseline to (1) evaluate subsequent progress in meeting current national^5^, ^40^, ^41^, ^42^ and global^2^ initiatives on public health targets associated with dementia care; and (2) facilitate cross‐national comparisons of good practice by translating successful approaches across different systems.

## CONFLICT OF INTEREST

None declared.

## AUTHOR CONTRIBUTIONS

Conception of this study: Linda Clare, Catherine Quinn, Rachel Collins. Data collection: IDEAL Co‐investigators. Data analysis and interpretation: Jayden O. van Horik. Drafting the article: Jayden O. van Horik. Critical revision of the article: Rachel Collins, Anthony Martyr, Catherine Henderson, Roy W. Jones, Martin Knapp, Catherine Quinn, Jeanette M. Thom, Christina Victor, Linda Clare. Final approval of the version to be published: Rachel Collins, Anthony Martyr, Catherine Henderson, Roy W. Jones, Martin Knapp, Catherine Quinn, Jeanette M. Thom, Christina Victor, Linda Clare.

## Supporting information

Supplementary Material

## Data Availability

IDEAL data were deposited with the UK data archive in April 2020 and will be available to access from April 2023. Details of how the data can be accessed after that date can be found here: http://reshare.ukdataservice.ac.uk/854293/.
