# Supplementary material for: Limited receipt of support services among people with mild‐to‐moderate dementia: Findings from the IDEAL cohort
Source: Int J Geriatr Psychiatry. 2022 Feb 6;37(3):10.1002/gps.5688. doi: 10.1002/gps.5688 (PMC9306706; doi:10.1002/gps.5688)
Supplement: Supplementary file 1 — Supplementary Material [file GPS-37-0-s001.docx]

**Supplementary Material**

**Limited receipt of support services among people with mild-to-moderate dementia: findings from the IDEAL cohort**

Jayden O. van Horik^a,b^, Rachel Collins^a^, Anthony Martyr^a^, Catherine Henderson^c^, Roy W Jones^d^, Martin Knapp^c^, Catherine Quinn^e,f^, Jeanette Thom^g^, Christina Victor^h^ and Linda Clare^a,i^, on behalf of the IDEAL Programme Team

^a^ REACH: The Centre for Research in Ageing and Cognitive Health, University of Exeter, Exeter, UK

^b^ Clinical Trials Unit, University of Exeter Medical School, Exeter, UK

^c^ Care Policy and Evaluation Centre, London School of Economics and Political Science, London, UK

^d^ RICE: The Research Institute for the Care of Older People, Royal United Hospitals, Bath, UK

^e^ The Centre for Applied Dementia Studies, University of Bradford, Bradford, UK

^f^ Wolfson Centre for Applied Health Research, Bradford, UK

^g^ School of Medical Sciences, University of New South Wales, Sydney, Australia

^h^ College of Health, Medicine and Life Sciences, Department of Health Sciences, Brunel University London, UK

^i^ NIHR Applied Research Collaboration South-West Peninsula, UK

**Table 1:** Support services questions asked of people living with dementia and informants.

**Tables 2-6:** Demographic measures retained in people living with dementia and informant minimal models (GLM) for receipt of respective support services.

**Table 1 SM.** Questions asked of people living with dementia and informants about support services

| **Question** |
| --- |
| **People living with dementia** |
| ***1a. Named health professional:*** *Is there a named health professional whom you can contact at any time, for example a care coordinator, key worker or specialist nurse?* |
| ***1b. Health professional in place due to dementia diagnosis:*** *Is the health professional available because of the dementia diagnosis?* |
| 1. ***Received information or educational materials:*** *Have you received any information or educational materials to help with your diagnosis?* 2. ***Interventions:*** *Have you taken part in any intervention (e.g. a support group, sessions with a psychologist, etc.) to help you with your diagnosis?* 3. ***Independently sought information:*** *Have you independently sought out information to help you with your diagnosis (e.g. have you contacted a national charity such as the Alzheimer's Society or Parkinson's UK, searched the internet for information, etc.)?* |
| **Informant** |
| ***1a. Named health professional:*** *Is there a named health professional whom you or your relative/friend can contact at any time, for example a care coordinator, key worker or specialist nurse?*  ***1b. Health professional in place due to dementia diagnosis:*** *Is the health professional in place to help with your relative's/friend's memory, thinking or behaviour difficulties?* |
| 1. ***Received information or educational materials:*** *Have you or your relative/friend with memory, thinking or behaviour difficulties received any information or educational materials to help support him/her with his/her memory, thinking or behaviour difficulties?* |
| 1. ***Interventions:*** *Has your relative/friend with memory, thinking or behaviour difficulties taken part in any intervention (such as a coping with memory difficulties group) to help with his/her memory, thinking or behaviour difficulties?* 2. ***Independently sought information:*** *Have you independently sought out information to help you support your relative/friend with memory, thinking or behaviour difficulties?* |

**Table 2 SM.** Demographic measures retained in people living with dementia and informant minimal models (GLM) for the *named health professional* question. Informant variables represent the characteristics of the person with dementia, not the informant. Degrees of freedom (DF), Odds Ratios and 95% Confidence Intervals (CI), z values and p values are presented. Significant measures (p < 0.05).

|  | **DF** | **Odds Ratio ± CI** | **z** | **p** |
| --- | --- | --- | --- | --- |
| **People living with dementia** |  |  |  |  |
| Sex: Female | 1 | 0.705 ± 0.243 | -2.539 | 0.011 |
| Age | 1 | 0.961 ± 0.014 | -5.646 | <0.001 |
| Educational Group: School leaving certificate at age 16 | 4 | 1.767 ± 0.347 | 3.213 | 0.001 |
| Educational Group: School leaving certificate at age 18 | 4 | 1.099 ± 0.312 | 0.595 | 0.552 |
| Educational Group: University | 4 | 1.120 ± 0.351 | 0.633 | 0.527 |
| Educational Group: Missing | 4 | 1.381 ± 0.933 | -0.048 | 0.962 |
| Diagnosis: Vascular dementia | 6 | 1.163 ± 0.378 | 0.780 | 0.435 |
| Diagnosis: Mixed Alzheimer’s disease/vascular dementia | 6 | 0.872 ± 0.310 | -0.860 | 0.390 |
| Diagnosis: Frontotemporal dementia | 6 | 1.330 ± 0.598 | 0.936 | 0.349 |
| Diagnosis: Parkinson’s disease dementia | 6 | 4.845 ± 0.653 | 4.733 | <0.001 |
| Diagnosis: Dementia with Lewy bodies | 6 | 2.935 ± 0.586 | 3.597 | <0.001 |
| Diagnosis: Unspecified/Other | 6 | 1.081 ± 0.710 | 0.215 | 0.830 |
| **Informant** |  |  |  |  |
| Sex: Female | 1 | 0.802 ± 0.259 | -1.674 | 0.094 |
| Age | 1 | 0.972 ± 0.016 | -3.544 | <0.001 |
| Carer Status: Family/Friend | 2 | 1.596 ± 0.339 | 2.699 | 0.007 |
| Diagnosis: Vascular dementia | 6 | 0.980 ± 0.398 | -0.099 | 0.921 |
| Diagnosis: Mixed Alzheimer’s disease/vascular dementia | 6 | 0.886 ± 0.312 | -0.759 | 0.448 |
| Diagnosis: Frontotemporal dementia | 6 | 1.011 ± 0.631 | 0.034 | 0.973 |
| Diagnosis: Parkinson’s disease dementia | 6 | 6.814 ± 0.794 | 4.736 | <0.001 |
| Diagnosis: Dementia with Lewy bodies | 6 | 1.828 ± 0.649 | 1.823 | 0.068 |
| Diagnosis: Unspecified/Other | 6 | 2.025 ± 0.745 | 1.859 | 0.063 |

**Table 3 SM.** Demographic measures retained in people living with dementia and informant minimal models (GLM) for the *health professional in place due to dementia diagnosis* question. Informant variables represent the characteristics of the person with dementia, not the informant. Degrees of freedom (DF), Odds Ratios and 95% Confidence Intervals (CI), z values and p values are presented. Significant measures (p < 0.05).

|  | **DF** | **Odds Ratio ± CI** | **z** | **p** |
| --- | --- | --- | --- | --- |
| **People living with dementia** |  |  |  |  |
| Age | 1 | 0.967 ± 0.022 | -3.147 | 0.002 |
| **Informant** |  |  |  |  |
| Age | 1 | 0.979 ± 0.027 | -1.563 | 0.118 |
| Carer Status: Family/Friend | 2 | 0.522 ± 0.537 | -2.367 | 0.018 |
| Diagnosis: Vascular dementia | 6 | 1.976 ± 0.821 | 1.626 | 0.104 |
| Diagnosis: Mixed Alzheimer’s disease/vascular dementia | 6 | 1.218 ± 0.572 | 0.675 | 0.500 |
| Diagnosis: Frontotemporal dementia | 6 | 1.028 ± 1.158 | 0.047 | 0.963 |
| Diagnosis: Parkinson’s disease dementia | 6 | 0.435 ± 0.784 | -2.079 | 0.038 |
| Diagnosis: Dementia with Lewy bodies | 6 | 2.193 ± 1.262 | 1.218 | 0.223 |
| Diagnosis: Unspecified/Other | 6 | 0.414 ± 1.043 | -1.656 | 0.098 |

**Table 4 SM.** Demographic measures retained in people living with dementia and informant minimal models (GLM) for the *received* *information or educational materials* question*.* Informant variables represent the characteristics of the person with dementia, not the informant. Degrees of freedom (DF), Odds Ratios and their 95% Confidence Intervals (CI), z values and p values are presented. Significant measures (p < 0.05).

|  | **DF** | **Odds Ratio ± CI** | **z** | **p** |
| --- | --- | --- | --- | --- |
| **People living with dementia** |  |  |  |  |
| Age | 1 | 0.945 ± 0.014 | -7.986 | <0.001 |
| Diagnosis: Vascular dementia | 6 | 1.295 ± 0.363 | 1.400 | 0.162 |
| Diagnosis: Mixed Alzheimer’s disease/vascular dementia | 6 | 1.020 ± 0.280 | 0.140 | 0.888 |
| Diagnosis: Frontotemporal dementia | 6 | 1.201 ± 0.633 | 0.565 | 0.572 |
| Diagnosis: Parkinson’s disease dementia | 6 | 3.717 ± 0.739 | 3.486 | <0.001 |
| Diagnosis: Dementia with Lewy bodies | 6 | 1.506 ± 0.598 | 1.342 | 0.180 |
| Diagnosis: Unspecified/Other | 6 | 0.685 ± 0.688 | -1.079 | 0.280 |
| **Informant** |  |  |  |  |
| Sex: Female | 1 | 0.747 ± 0.261 | -2.189 | 0.029 |
| Diagnosis: Vascular dementia | 6 | 1.209 ± 0.435 | 0.855 | 0.393 |
| Diagnosis: Mixed Alzheimer’s disease/vascular dementia | 6 | 1.341 ± 0.343 | 1.679 | 0.093 |
| Diagnosis: Frontotemporal dementia | 6 | 1.781 ± 0.829 | 1.365 | 0.172 |
| Diagnosis: Parkinson’s disease dementia | 6 | 0.350 ± 0.631 | -3.263 | 0.001 |
| Diagnosis: Dementia with Lewy bodies | 6 | 0.695 ± 0.688 | -1.036 | 0.300 |
| Diagnosis: Unspecified/other | 6 | 0.971 ± 0.833 | -0.070 | 0.944 |

**Table 5 SM.** Demographic measures retained in people living with dementia and informant minimal models (GLM) for the *interventions* question*.* Informant variables represent the characteristics of the person with dementia, not the informant. Degrees of freedom (DF), Odds Ratios and their 95% Confidence Intervals (CI), z values and p values are presented. Significant measures (p < 0.05).

|  | **DF** | **Odds Ratio ± CI** | **z** | **p** |
| --- | --- | --- | --- | --- |
| **People living with dementia** |  |  |  |  |
| Sex: Female | 1 | 0.743 ± 0.251 | -2.311 | 0.020 |
| Age | 1 | 0.979 ± 0.014 | -2.922 | 0.003 |
| Carer Status: Family/Friend | 2 | 0.996 ± 0.370 | -0.020 | 0.984 |
| Carer Status: No carer involved | 2 | 0.695 ± 0.347 | -2.051 | 0.040 |
| Diagnosis: Vascular dementia | 6 | 0.992 ± 0.392 | -0.040 | 0.968 |
| Diagnosis: Mixed Alzheimer’s disease/vascular dementia | 6 | 0.990 ± 0.306 | -0.064 | 0.949 |
| Diagnosis: Frontotemporal dementia | 6 | 0.864 ± 0.662 | -0.430 | 0.667 |
| Diagnosis: Parkinson’s disease dementia | 6 | 1.554 ± 0.647 | 1.337 | 0.181 |
| Diagnosis: Dementia with Lewy bodies | 6 | 1.067 ± 0.625 | 0.202 | 0.840 |
| Diagnosis: Unspecified/other | 6 | 0.969 ± 0.725 | -0.085 | 0.932 |
| **Informant** |  |  |  |  |
| Educational Group: School leaving certificate at age 16 | 4 | 0.828 ± 0.386 | -0.956 | 0.339 |
| Educational Group: School leaving certificate at age 18 | 4 | 1.486 ± 0.308 | 2.521 | 0.012 |
| Educational Group: University | 4 | 1.177 ± 0.357 | 0.895 | 0.371 |
| Educational Group: Missing | 4 | 1.063 ± 0.759 | -0.044 | 0.965 |
| Diagnosis: Vascular dementia | 6 | 1.233 ± 0.392 | 1.048 | 0.295 |
| Diagnosis: Mixed Alzheimer’s disease/vascular dementia | 6 | 1.461 ± 0.302 | 2.465 | 0.014 |
| Diagnosis: Frontotemporal dementia | 6 | 1.446 ± 0.627 | 1.154 | 0.249 |
| Diagnosis: Parkinson’s disease dementia | 6 | 0.303 ± 0.951 | -2.463 | 0.014 |
| Diagnosis: Dementia with Lewy bodies | 6 | 1.543 ± 0.647 | 1.314 | 0.189 |
| Diagnosis: Unspecified/Other | 6 | 0.701 ± 0.876 | -0.795 | 0.427 |

**Table 6 SM.** Demographic measures retained in people living with dementia and informant minimal models (GLM) for the *independently sought information* question*.* Informant variables represent the characteristics of the person with dementia, not the informant. Degrees of freedom (DF), Odds Ratios and their 95% Confidence Intervals (CI), z values and p values are presented. Significant measures (p < 0.05).

|  | **DF** | **Odds Ratio ± CI** | **z** | **p** |
| --- | --- | --- | --- | --- |
| **People living with dementia** |  |  |  |  |
| Sex: Female | 1 | 0.742 ± 0.264 | -2.203 | 0.028 |
| Age | 1 | 0.939 ± 0.016 | -7.825 | <0.001 |
| Marital Status: Married/Partnership/Cohabiting | 3 | 0.771 ± 0.976 | -0.521 | 0.602 |
| Marital Status: Divorced/Legally separated | 3 | 1.368 ± 1.066 | 0.576 | 0.564 |
| Marital Status: Widowed | 3 | 0.591 ± 1.027 | -1.002 | 0.316 |
| Educational Group: School leaving certificate at age 16 | 4 | 1.626 ± 0.394 | 2.419 | 0.016 |
| Educational Group: School leaving certificate at age 18 | 4 | 1.724 ± 0.339 | 3.152 | 0.002 |
| Educational Group: University | 4 | 2.691 ± 0.367 | 5.306 | <0.001 |
| Educational Group: Missing | 4 | 0.490 ± 2.225 | -0.630 | 0.529 |
| Diagnosis: Vascular dementia | 6 | 1.047 ± 0.408 | 0.221 | 0.825 |
| Diagnosis: Mixed Alzheimer’s disease/vascular dementia | 6 | 0.945 ± 0.325 | -0.344 | 0.731 |
| Diagnosis: Frontotemporal dementia | 6 | 1.127 ± 0.657 | 0.357 | 0.721 |
| Diagnosis: Parkinson’s disease dementia | 6 | 4.687 ± 0.710 | 4.271 | <0.001 |
| Diagnosis: Dementia with Lewy bodies | 6 | 2.041 ± 0.617 | 2.266 | 0.023 |
| Diagnosis: Unspecified/Other | 6 | 0.816 ± 0.753 | -0.528 | 0.598 |
| **Informant** |  |  |  |  |
| Sex: Female | 1 | 0.757 ± 0.259 | -2.100 | 0.036 |
| Age | 1 | 0.958 ± 0.016 | -5.273 | <0.001 |
| Carer Status: Family/Friend | 2 | 2.312 ± 0.351 | 4.685 | <0.001 |
| Carer Status: No carer involved | 2 | 3.486 ± 0.636 | 0.039 | 0.969 |
| Educational Group: School leaving certificate at age 16 | 4 | 0.960 ± 0.355 | -0.225 | 0.822 |
| Educational Group: School leaving certificate at age 18 | 4 | 1.344 ± 0.308 | 1.888 | 0.059 |
| Educational Group: University | 4 | 1.781 ± 0.359 | 3.153 | 0.002 |
| Educational Group: Missing | 4 | 4.395 ± 1.486 | -1.084 | 0.278 |
